# 5p13.3p13.2 duplication associated with developmental delay, congenital malformations and chromosome instability manifested as low-level aneuploidy

**DOI:** 10.1186/s40064-015-1399-3

**Published:** 2015-10-15

**Authors:** Ivan Y. Iourov, Svetlana G. Vorsanova, Irina A. Demidova, Galina A. Aliamovskaia, Elena S. Keshishian, Yuri B. Yurov

**Affiliations:** Mental Health Research Center, Russian Academy of Medical Sciences, Moscow, 117152 Russia; Russian National Research Medical University named after N.I. Pirogov, Separated Structural Unit “Clinical Research Institute of Pediatrics”, Ministry of Health of Russian Federation, Moscow, 125412 Russia; Department of Medical Genetics, Russian Medical Academy of Postgraduate Education, Moscow, 123995 Russia

**Keywords:** 5p13 duplication, Chromosome instability, Aneuploidy, Mosaicism, Bioinformatics

## Abstract

Recent developments in molecular cytogenetics allow the detection of genomic rearrangements at an unprecedented level leading to discoveries of previously unknown chromosomal imbalances (zygotic and post-zygotic/mosaic). These can be accompanied by a different kind of pathological genome variations, i.e. chromosome instability (CIN) manifested as structural chromosomal rearrangements and low-level mosaic aneuploidy. Fortunately, combining whole-genome and single-cell molecular cytogenetic techniques with bioinformatics offers an opportunity to link genomic changes to specific molecular or cellular pathology. High-resolution chromosomal SNP microarray analysis was performed to study the genome of a 15-month-aged boy presented with developmental delay, congenital malformations, feeding problems, deafness, epileptiform activity, and eye pathology. In addition, somatic chromosomal mutations (CIN) were analyzed by fluorescence in situ hybridization (FISH). Interstitial 5p13.3p13.2 duplication was revealed in the index patient. Moreover, CIN manifested almost exclusively as chromosome losses and gains (aneuploidy) was detected. Using bioinformatic analysis of SNP array data and FISH results, CIN association with the genomic imbalance resulted from the duplication was proposed. The duplication was demonstrated to encompass genes implicated in cell cycle, programmed cell death, chromosome segregation and genome stability maintenance pathways as shown by an interactomic analysis. Genotype-phenotype correlations were observed, as well. To the best our knowledge, identical duplications have not been reported in the available literature. Apart from genotype-phenotype correlations, it was possible to propose a link between the duplication and CIN (aneuploidy). This case study demonstrates that combining SNP array genomic analysis, bioinformatics and molecular cytogenetic evaluation of somatic genome variations is able to provide a view on cellular and molecular pathology in a personalized manner. Therefore, one can speculate that similar approaches targeting both interindividual and intercellular genomic variations could be useful for a better understanding of disease mechanisms and disease-related biological processes.

## Background

During the last decade, molecular cytogenetics has made significant efforts towards the development of new technologies, which are able to detect genomic rearrangements at an unprecedented resolution (Yan et al. 2009; Carvill and Mefford 2013; Kloosterman and Hochstenbach 2014; Riegel 2014). As a result, a broad spectrum of previously unreported microdeletion and microduplication syndromes has been discovered (Weise et al. 2012; Carvill and Mefford 2013). Additionally, numerous unique chromosomal (subchromosomal) abnormalities have been described providing important clues regarding the origins and consequences of genomic rearrangements (Kloosterman and Hochstenbach 2014; Riegel 2014). Interestingly, both single gene mutations and copy number variations of chromosomal DNA (deletions/duplications) affecting almost all cells of the body (non-mosaic/heritable genetic changes), are able to produce genome or chromosome instability (CIN) manifesting as structural or numerical chromosomal changes (Thompson et al. 2010; Yurov et al. 2011; Forsberg et al. 2013; Heng et al. 2013; Iourov et al. 2013b; Pine and Liu 2014). CIN leading to the propagation of aneuploid cells represents an important phenomenon, which mediates intercellular genetic diversity in health and disease hallmarking cancers, neurodegeneration and aging (Michor et al. 2005; Iourov et al. 2009; Thompson et al. 2010; Vorsanova et al. 2010a; Yurov et al. 2011; Abdallah et al. 2013; Forsberg et al. 2013; Hultén et al. 2013). In this context, descriptions of cases featured by non-mosaic genetic changes and exhibiting increased levels of somatic mutations or CIN seem to be of fundamental importance (Michor et al. 2005; Iourov et al. 2013b).

Here, we report a male child with developmental delay, severe congenital malformations and interstitial duplication at 5p13.3p13.2, who has also exhibited increased levels of sporadic aneuploidy (CIN). Application of high-resolution chromosomal SNP microarray analysis, fluorescence in situ hybridization (FISH) and bioinformatics has allowed speculations about correlations between genotype and phenotype. Furthermore, interactomic analysis has shown that CIN is likely to be associated with the duplication through a slight change of cell cycle, chromosome segregation, programmed cell death and genome stability maintenance pathways.

## Case description

The male patient was referred to the hospital at the age of 15 months because of a developmental delay, congenital malformations, blindness, and feeding problems (poor appetite and frequent regurgitation). The familial history is positive for chronic gastritis and congenital heart malformation (mother), extensive brain hemorrhage (cousin; died at the ninth day postpartum) on the maternal side and congenital lung malformation (uncle; died at the first day postpartum), pancreatic cancer (grandfather) on the paternal side. The patient was born to a primigravida at 34th week of gestation by cesarean section. The pregnancy was threatened by miscarriage and was complicated by a low grade fever and exacerbation of gastritis (14th–17th weeks). Ultrasound showed oligohydramnios and fetal growth delay. The parents were non-consanguineous. The birth weight was 1297 g and birth length was 38 cm (both significantly less than 3rd percentile for age). Apgar score was 1 at 1 min and 4 at 5 min. He had several respiratory failures and required intubation and intermittent positive pressure ventilation for 20 days. A diffuse hemorrhagic rash was noted at the first days of life. He had feeding problems, i.e. regurgitations persisting over a long period of time. Growth retardation was observed during the first year of life (always less than 3rd percentile for age). A developmental delay was also noted. The child did not recognize mother’s face and was uncommunicative. Emotional response and object interests were lacking. At the age of 1 year, sensorineural deafness was revealed. Focal epileptiform activity in the left parietal-occipital region was also reported.

Physical examination at 15 months revealed a significant deficit in weight and decreased head circumference (less than 3rd percentile). He presented with dolichomicrocephaly, high forehead, ocular hypotelorism (the inner and outer canthal distances were both less than 3rd percentile), epicanthus, long philtrum, low set auricles, and enamel hypoplasia. Mild left-sided spastic hemiparesis and muscular hypotonia were noted. The psychomotor development (CAT/CLAMS scale) corresponded to the age of 5–6 months; the developmental coefficients were less than 50 %. He demonstrated stereotypic tapping of his head by his arm. The abdominal ultrasound examinations showed mild splenomegaly and increased echogenicity of parenchyma. The ophthalmological investigation showed descending optic atrophy, concomitant strabismus, nystagmus, zonular cataract of the left eye. Corpus callosum hypoplasia was uncovered by magnetic resonance imaging.

**Fig. 1:**
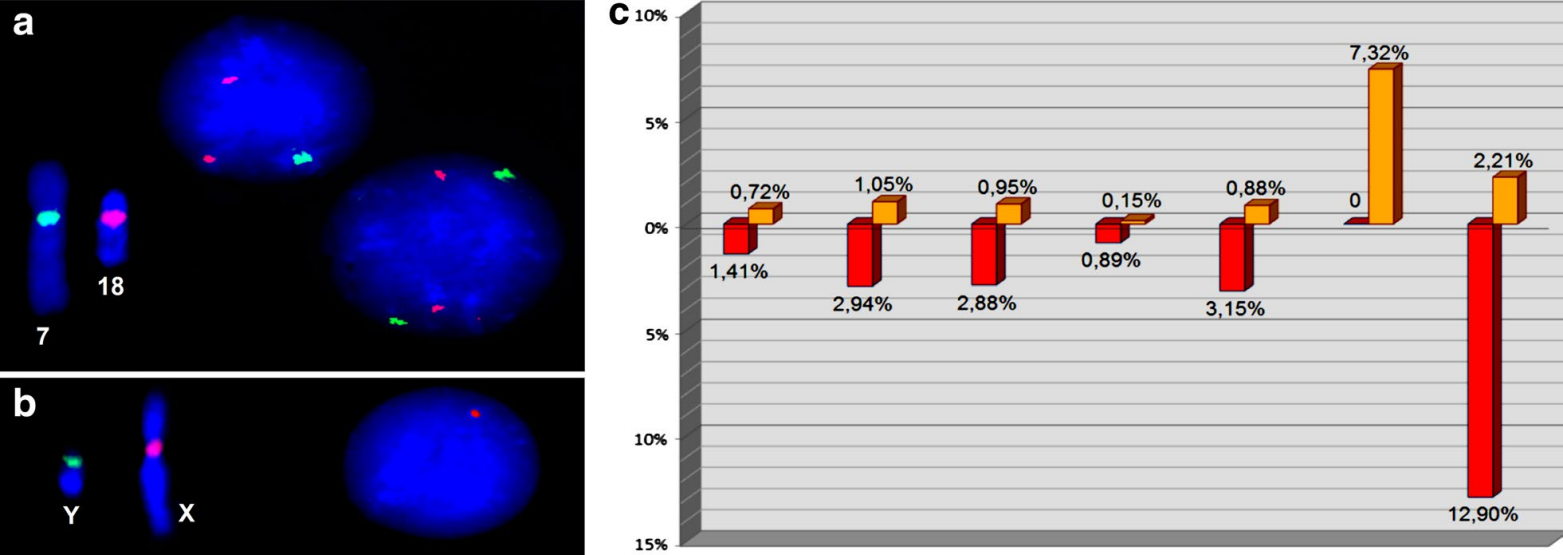
Interphase FISH analysis of CIN (somatic aneuploidy). **a** FISH with DNA probes for chromosomes 7 (*green*) and 18 (*red*) showing chromosome 7 loss in the right nucleus (metaphase chromosomes show positive signals for these DNA probes). **b** Interphase FISH with DNA probes for chromosomes Y (*green*) and X (*red*) showing chromosome Y loss in the nucleus (metaphase chromosomes show positive signals for these DNA probes). **c** Rates of chromosome losses (*red bars*) and gains (*golden bars*)

Cytogenetic analysis was performed by GTG- (~500 band resolution) and C-banding and showed the presence of CIN (mainly chromosome gains or losses—aneuploidy). Constitutional chromosomal imbalances were not detected. Parental karyotypes were normal. To determine CIN rates, we performed FISH with DNA probes for chromosomes 1, 7, 16, 17, 18, X and Y as described earlier (Yurov et al. 2007; Iourov et al. 2009; Vorsanova et al. 2010b). FISH analysis confirmed the presence of CIN (aneuploidy) and showed that the rate of somatic/sporadic chromosomal mutations is 3-7 times higher in the index patient (Fig. 1) as to average chromosomal mutation rates in the general population according to our previous studies (Yurov et al. 2007; Iourov et al. 2009; Vorsanova et al. 2010a; Hultén et al. 2013).

**Fig. 2:**
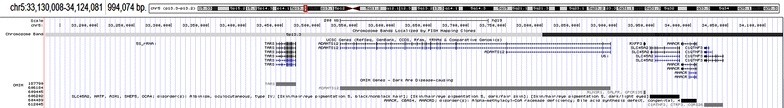
Schematic representation of 5p13.3p13.2 duplication detected by high-resolution chromosomal microarray analysis displayed using UCSC Genome Browser on Human Feb. 2009 Q2 381 (GRCh37/hg19) Assembly (http://genome-euro.ucsc.edu/index.html)

High-resolution chromosomal SNP microarray analysis was performed on DNA isolated from peripheral blood lymphocytes using CytoScan^®^ HD Arrays (Affymetrix, Santa Clara, CA) consisting of ~2.7 million markers for CNV evaluation and ~750,000 SNPs and the Affymetrix Chromosome Analysis Suite software as described earlier (Iourov et al. 2012, 2013a; Wang et al. 2014). Genomic localization and gene content was defined using NCBI Build GRCh37/hg19 reference sequence. Genomic copy number variations were further addressed by a bioinformatic analysis described recently (Iourov et al. 2014). Interactomic analysis was performed using STRING v9.1 online tool (Franceschini et al. 2013). High-resolution chromosomal SNP microarray analysis has detected a duplication located at 5p13.3p13.2 (~994 kbps) spanning 11 genes, 6 of which have assigned functions and are listed in OMIM (Online Mendelian Inheritance in Men) database (http://omim.org/) (Fig. 2). The duplication was *de novo*.

According to microarray analysis (Fig. 2), 6 genes were found to be eligible for bioinformatic evaluation. *TARS* (OMIM:#187790) encodes an aminoacyl-tRNA synthetase interacting with numerous proteins from this family. Secondary functions of *TARS* are shown to be associated with the nervous system-related disorders and autoimmune diseases (Williams et al. 2013). *ADAMTS12* (OMIM:#606184) encodes a member of a disintegrin and metalloproteinase with thrombospondin motifs protein family with tumour-protective functions (Llamazares et al. 2007) interacting with a number of gene products, among which there are proteins implicated in Notch signaling pathway (*NOTCH1*, *FURIN*). Functionally, *ADAMTS12* copy number variation has the potential to be relevant to numerous pathogenic processes including those associated with brain dysfunction, autoimmunity, inflammation, and cancer. *RXFP3* (OMIM:#609445) is a member of relaxin family peptide receptors. Bioinformatic analysis shows that copy number variation of *RXFP3* may alter neuropeptide signaling processes, organization of neuroendocrine signals and stimulation of water drinking and food intake. *SLC45A2* (OMIM:#606202) encodes a transporter protein mediating melanin synthesis and is mutated in oculocutaneous albinism type IV (OMIM:#606574). According to BioGPS, this gene is expressed in retina (http://biogps.org/#goto=genereport&id=51151). *AMACR* (OMIM:#604489) encodes a racemase and is likely to be mutated in adult-onset sensorimotor neuropathy, pigmentary retinopathy, and adrenomyeloneuropathy due to defects in bile acid synthesis. In addition, the gene product interacts with multifunctional proteins, which play a role in transcriptional and cell cycle regulation, programmed cell death, and genome stability maintenance (Lloyd et al. 2012). *C1QTNF3* (OMIM:#612045) is a gene encoding C1q and tumor necrosis factor related protein 3 mutated in neurobehavioral diseases. The gene product interacts with leptin, an element of a signaling pathway that acts to regulate the size of the body, fat depot and may act on the brain to inhibit food intake. Bioinformatic analysis has given rise to speculations that this duplication is likely to be causative for developmental delay and congenital malformations in the index case. Moreover, it allowed us to determine the molecular pathway to CIN, the elements of which were involved in the duplication.

## Discussion and evaluation

During the last decades, there have been several reports on duplications at 5p13 (Lorda-Sánchez et al. 1997; Cervera et al. 2005; Loscalzo et al. 2008). Further molecular cytogenetic studies have evidenced that similar duplications cause chromosome 5p13 duplication syndrome (OMIM #613174) involving the *NIPBL* gene. Clinically, the syndrome is characterized by developmental delay and intellectual disability with facial dysmorphism featured by frontal bossing, large or broad forehead, bitemporal narrowing, short/slanted palpebral fissures, short philtrum, high-arched palate, and low-set ears (Yan et al. 2009). However, the index case has not demonstrated duplication of the critical region of the chromosome 5p13 duplication syndrome. Nevertheless, molecular and clinical overlap between classical chromosome 5p13 duplication syndrome and the index case was noted. Interestingly, two cases (patients: 286710 and 300639) from DECIPHER v8.8 (https://decipher.sanger.ac.uk) exhibited duplications encompassing shortly genomic loci affected by the duplication in the index patient. Although patient 300639 presented with global developmental delay, both cases seem to demonstrate a milder phenotype compared to the present case. Nonetheless, it is to note that these cases were not identical to the present one. The phenotypic differences are likely to be explained by incomplete overlap of duplicated chromosomal DNA sequences between DECIPHER cases and the index patient. To provide case-specific genotype-phenotype correlations, we have used a recently described bioinformatic algorithm for molecular cytogenetic analysis (Iourov et al. 2014).

Bioinformatic evaluation of duplicated genes enabled us to speculate on genotype-phenotype correlations. Thus, copy number increase of *TARS* is likely to be related to developmental delay and congenital malformations; *ADAMTS12*—brain dysfunction, developmental delay and congenital malformations; *RXFP3*—neurological symptoms and feeding problems; *SLC45A2*—eye pathology; *AMACR*—neurological symptoms (i.e. deafness), eye pathology and congenital malformations; *C1QTNF3*—growth retardation and feeding problems. Although in silico evaluation allowed genotype-phenotype correlations in the same fashion as done for almost all contiguous gene deletion/duplication syndromes (Yan et al. 2009; Weise et al. 2012; Kloosterman and Hochstenbach 2014; Carvill and Mefford 2013), there can be a certain bias and cross effects between phenotypic contribution of each duplicated gene. Surprisingly, the phenotype of the duplication was rather severe. Still, bioinformatics analysis, genotype-phenotype correlations and comparative evaluation with previous reports on chromosome 5p13 duplication syndrome do indicate a possible causative role of this genomic rearrangement.

**Fig. 3:**
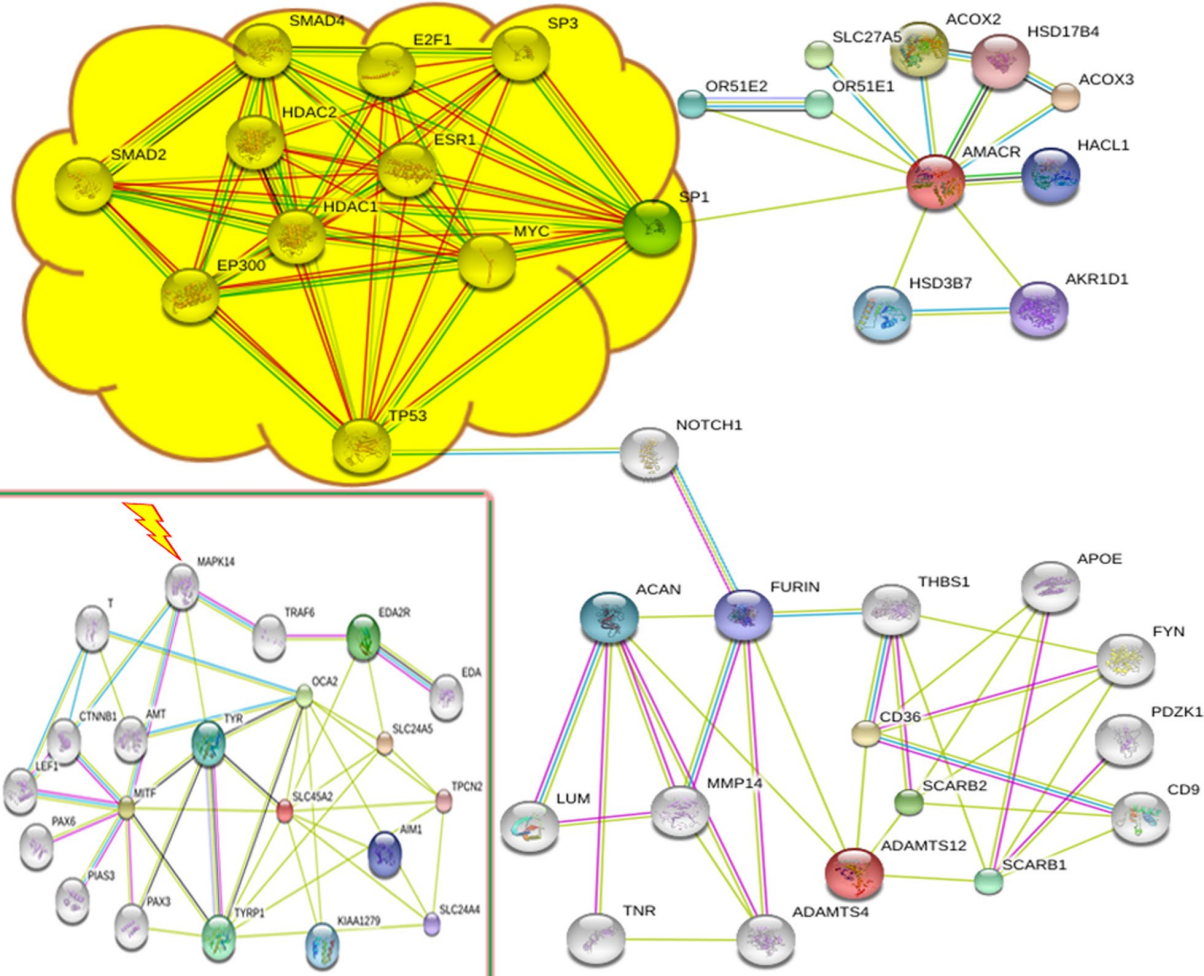
Shared interactome of *AMCAR* and *ADAMTS12* (note the part of the interactome indicated by a *yellow cloud*, which is related to “aneuploidization” pathway) and interactome of *SLC45A2* (note the presence of *MAPK14* indicated by a *lightning bolt*); *AMCAR*, *ADAMTS12* and *SLC45A2* are coloured *red*; interactomes are depicted using STRING v9.1 online tool (Franceschini et al. 2013)

It is noteworthy that *ADAMTS12*, *AMACR*, *SLC45A2* and *C1QTNF3* are associated with cancers, which are all hallmarked by CIN (aneuploidy) (Michor et al. 2005; Llamazares et al. 2007; Lloyd et al. 2012; Abdallah et al. 2013). Somatic aneuploidy is generally a result of alterations to cell cycle checkpoint, chromosome segregation, programmed cell death and genome stability maintenance pathways, which can be fused into a single generalized “aneuploidization” pathway (Harris and Levine 2005; Iourov et al. 2009, 2013b; Yurov et al. 2011; Abdallah et al. 2013; Heng et al. 2013). Interactome analysis of proteins encoded by duplicated genes provided a possibility to link the genomic rearrangement and CIN (Fig. 3). It has been observed that *ADAMTS12* and *AMACR* interactomes are interconnected via *NOTCH1* and *TP53*. These are key elements of global pathways of cell cycle/fate and transcriptional activity regulation. Furthermore, *NOTCH1* and *TP53* are together involved in pathways leading to CIN in a variety of cancers (Roemer 2012; Pine and Liu 2014). According to the analysis, the connection between *ADAMTS12* and *AMACR* interactomes implicate numerous elements of the critical pathways, which were previously grouped under the umbrella term “aneuploidzation” pathway (Iourov et al. 2009, 2013b; Yurov et al. 2011; Abdallah et al. 2013; Heng et al. 2013). In this light, it is to mention *MAPK14* as an element of *SLC45A2* interactome (Fig. 3), inasmuch as *MAPK14* gene product has been shown to play a role in progression of *TP53*(−/−) cells exhibiting numerical chromosome abnormalities and CIN (Vitale et al. 2010). It is to recognize that duplicated genes are indirectly involved in the aforementioned pathway. Taking this fact into account, we admit that CIN might be a result of another genetic or epigenetic change. However, since CIN in the index patient is limited to increase of somatic aneuploidy rates being significantly milder than in a cell population exhibiting alterations to the genes directly implicated in the aforementioned pathway (i.e. *TP53*, *EP300*, *SP1* etc.) (Thompson et al. 2010; Roemer 2012; Heng et al. 2013; Pine and Liu 2014). Consequently, we have hypothesized that alterations to less essential neighbors in an interactome network are more likely to result into a slight modification of the pathway. The latter can produce specific types of CIN without an immediate impact on cellular genotype/phenotype. Additionally, CIN affecting detectable cell populations should be considered a potential risk factor for disorders mediated by genome instability (i.e. cancer and neurodegeneration) requiring molecular cytogenetic monitoring of somatic chromosomal mutations. To this end, we have to state that a relationship between CIN and 5p13.3p13.2 duplications requires additional case-reports as well as additional molecular analyses.

## Conclusion

In the present case study, a description of 5p13.3p13.2 duplication in a child with developmental delay, congenital malformations and CIN (sporadic aneuploidy) is given. Using high-resolution chromosomal SNP microarray analysis, FISH evaluation of somatic aneuploidy and bioinformatics, we were able to propose genotype-phenotype correlation and to hypothesize a link between non-mosaic 5p13 duplication and sporadic mosaic aneuploidy (CIN) in the index case. Accordingly, we speculate that combining techniques targeting interindividual and intercellular genome variations together with bioinformatic analyses is able to uncover underlying basis of phenotypic changes due to genomic rearrangements or disease mechanisms in a personalized manner.

## Abbreviations

CIN: chromosome instability
FISH: fluorescence in situ hybridization
SNP: single nucleotide polymorphism
